# Beating aortic valve replacement surgery as an alternative to transcatheter aortic valve implantation in a patient with severe aortic stenosis and left ventricular dysfunction

**DOI:** 10.1186/s13019-018-0818-2

**Published:** 2018-12-27

**Authors:** Kenji Iino, Yoshitaka Yamamoto, Hideyasu Ueda, Hirofumi Takemura

**Affiliations:** Department of Thoracic, Cardiovascular and General Surgery, Kanazawa University, 13-1 Takaramachi, Kanazawa, 920-8641 Japan

**Keywords:** Aortic stenosis, Bicuspid valve, Aortic valve replacement

## Abstract

**Background:**

Transcatheter aortic valve implantation (TAVI) is the standard treatment for high-risk patients with aortic stenosis (AS); however, alternative treatments for patients who are ineligible for TAVI are controversial.

**Case presentation:**

56 year-old female who required 6 γ dobutamine support due to congestive heart failure was diagnosed as severe aortic stenosis with bicuspid valve. Echocardiography revealed left ventricular ejection fraction (LVEF) was 15%. The patient was relatively young for TAVI, and TAVI was not licensed for patient presenting with a bicuspid aortic valve in places other than the limited institutions in Japan. On pump beating aortic valve replacement (AVR) was performed with selective antegrade coronary artery blood perfusion. She resumed a completely normal lifestyle by 3 weeks after the operation.

**Conclusions:**

A relatively young patient for TAVI who was diagnosed as aortic stenosis with severely reduced ejection fraction and bicuspid valve is reported. Beating AVR with a continuously selective antegrade-perfusion was achieved safely with good clinical results in a patient with severely reduced left ventricular (LV) function. Beating AVR can be considered as a potential alternative for patients who are ineligible for conventional surgical aortic valve replacement (SAVR) and TAVI.

## Background

TAVI has shown excellent results in inoperable and high-risk patients who are at very high risk of adverse outcome from conventional SAVR [1, 2]. However, some patients are ineligible for TAVI because of anatomical contraindications or uncertainty with regard to its long-term durability [3]. The current report describes a case of successful beating aortic valve replacement (AVR) in a patient with severely impaired LV systolic function, who was ineligible for TAVI.

## Case presentation

A 56-year-old woman presented to our hospital with acute congestive heart failure. She needed dobutamine support and bilevel positive airway pressure for NYHA class IV dyspnea. Chest radiography revealed congestive heart failure. Echocardiography revealed severe aortic stenosis with heavily calcific bicuspid valve; the LVEF was significantly reduced at 15%. The aortic valve area measured 0.52 cm^2^. Mean pressure gradient was 49 mmHg. A peak aortic jet velocity was 4.4 m/s. Right-heart catheterization revealed a cardiac index of 1.6 L/min/m^2^ and pulmonary hypertension with the mean pulmonary artery pressure of 55 mmHg. Coronary angiography showed normal coronary vasculature without signs of significant stenosis. The pulmonary capillary wedge pressure was 37 mmHg. Computed tomography demonstrated a mildly dilated ascending aorta with a diameter of 42 mm. She was diagnosed as heart failure reduced ejection fraction (HFrEF) due to severe aortic stenosis. The society of Thoracic Surgeons predicted mortality score was 12.4%.

Our heart team discussed her treatment. Our patient was a younger AS patient with severe LV contractile dysfunction and with bicuspid valve. Considering the severe LV contractile dysfunction, the patient seemed suitable for TAVI as the lack of ischemic cardiac arrest and extracorporeal circulation helps avoid ischemia, as well as ischemic reperfusion injury, inflammatory reaction, and oxidative stress. However, we hesitated to perform TAVI for this patient because extension of TAVI to such a younger patient with longer life-expectancy raises the issue of durability. Leaflet asymmetry of the implanted transcatheter heart valve which might occur after deployment into bicuspid valve may have an impact on long-term valve durability. While, conventional SAVR is possible while the heart is arrested with cardioplegic arrest, which is effective in majority of AS patients with acceptable morbidity and mortality. However, in some cases, especially in patients with impaired LV function like our patient, ischemic period followed by reperfusion period may lead to myocardial injury, which is associated with high perioperative mortality and morbidity. If SAVR could be performed with beating heart condition, the patient had benefited from this procedure without myocardial ischemia similar to TAVI. In spite of recent advances in myocardial protection methods, blood supply is the most effective technique of myocardial protection under beating heart condition. Cardioplegic arrest may induce reperfusion injury. In contrast, maintaining the myocardial contraction results in less myocardial edema and better cardiac function [4]. We therefore decided to perform on-pump beating AVR with selective antegrade coronary artery blood perfusion.

**Fig. 1:**
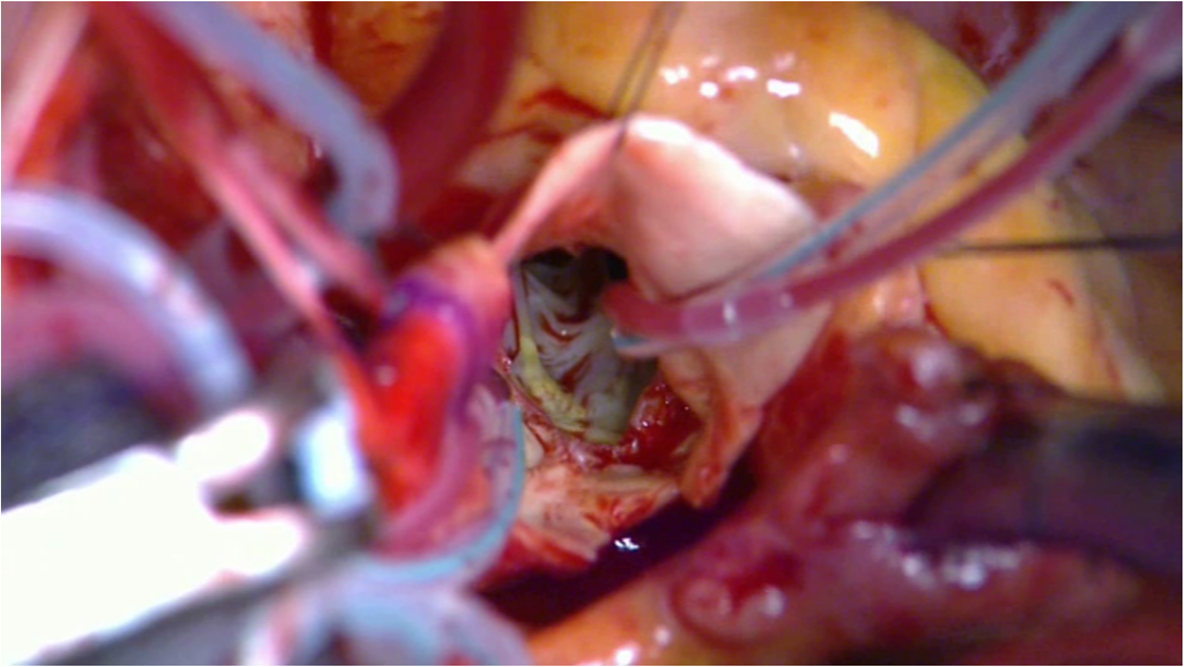
On-pump beating aortic valve replacement with selective antegrade coronary artery blood perfusion

She could not lie on her back due to severe orthopnea. Therefore, percutaneous cardiopulmonary support (PCPS) was initiated at the femoral vessels before the induction of general anesthesia. Surgery was performed via a median sternotomy. Cardiopulmonary bypass (CPB) was established after central cannulation. Under systemic temperature of 35–37 °C, CPB flow rates maintained at 2.5–2.8 L/min/m^2^, with a mean systemic pressure 60–80 mmHg. The aorta was crossed-clamped and opened. Direct 5-Fr silicon coronary perfusion cannulas (Sumitomo Bakelite, Tokyo, Japan) were inserted into the left and right coronary ostia, and oxygenated blood was continuously perfused at 34 °C and at a rate of 300 ml/min with mean perfusion pressure of 150 mmHg. The cannulas were fixed to the aortic wall with 5–0 prolene, and they were secured by tying them to a tourniquet. The valve was a severe calcific true bicuspid valve. Calcified leaflets were removed using the usual approach. The calcifications extending to the aortic annulus were carefully removed using a SONOPET ultrasonic aspirator (Stryker, Kalamazoo, MI) and scalpel (Fig. 1). (Video). After sizing the annulus, a 21-mm Regent mechanical valve (St. Jude Medical, St. Paul, MN) was placed into the aortic annulus using continuous suture technique with three 2–0 prolene sutures. The aorta was closed using the standard technique. Horizontal mattress suturing was performed for the first layer. After de-airing of the left ventricle, the aortic clamp was removed. Running suturing was performed for the second layer to ensure hemostasis. Transesophageal echocardiography was used to access septal and ventricular wall motion during surgery. The patient was weaned off CPB and PCPS under intra-aortic balloon pumping (IABP) support. Post-operative echocardiography demonstrated improved wall motion and an increase in the ejection fraction of up to 40%. She resumed a completely normal lifestyle by 3 weeks after the operation.

## Discussion

Aortic outflow obstruction relieved mechanically via SAVR can significantly improve symptoms, LV function, and survival in patients with severe aortic stenosis [5]. However, the outcome of SAVR is largely dependent on pre-operative LV function [6]. In patients with impaired LVEF, SAVR is associated with high perioperative mortality and morbidity [5, 7].

TAVI has become a standard treatment for high-risk patients with aortic stenosis who are not considered suitable for SAVR owing to increased surgical risk [1, 2]. Moreover, TAVI is associated with greater LVEF improvement compared with SAVR in patients with severely depressed LV function, as the lack of ischemic cardiac arrest and extracorporeal circulation helps avoid ischemia, as well as ischemic reperfusion injury, inflammatory reaction, and oxidative stress, which can cause apoptosis and contractile dysfunction in surviving myocytes [8]. The current case was considered suitable for TAVI, especially considering that TAVI is associated with favorable effects with regard to LVEF recovery. However, we did not perform TAVI in our relatively young patient because the long-term durability of TAVI remains unknown and leaflet asymmetry of the implanted transcatheter heart valve which might occur after deployment into bicuspid valve may have an impact on long-term valve durability.

Alternative treatments for patients who are ineligible for TAVI are controversial. Beating AVR has been reported as an alternative procedure to conventional AVR in patients with aortic stenosis after previous coronary artery bypass surgery for reducing the risk of patent internal mammary artery graft injury [9–11]. Various myocardial perfusion techniques have been described using antegrade perfusion through the internal mammary artery (IMA), venous bypass graft or retrograde coronary sinus perfusion, or both [9–11]. As our patient didn’t have previous CABG history, myocardial perfusion was continuously performed through direct 5-Fr silicon coronary perfusion cannulas into the left and right native coronary ostia and was totally dependent on this antegrade continuous coronary perfusion. As beating AVR with antegrade blood perfusion has the advantage of maintaining the physiological condition of the heart throughout the procedure, it is an alternative surgical option for high-risk patients with impaired LV function. However, in case the surgeon cannot expose adequately the aortic annulus, or keep the coronary perfusion cannulas in place, the surgeon may abandon beating AVR with antegrade perfusion and convert to cardioplegic arrest. It is necessary to decide which strategy is the most beneficial for individual patient by considering cardiac function, degree of calcification of the ascending aorta, and condition of the native coronary artery.

## Conclusions

Beating AVR with a continuously selective antegrade-perfusion was achieved safely with good clinical results in a patient with severely reduced LV function. Beating AVR can be considered as a potential alternative for patients who are ineligible for conventional SAVR and TAVI.

## Abbreviations

AS: Aortic stenosis
AVR: Aortic valve replacement
CPB: Cardiopulmonary bypass
HFrEF: Heart failure reduced ejection fraction
IABP: Intra-aortic balloon pumping
IMA: Internal mammary artery
LV: Left ventricular
LVEF: Left ventricular ejection fraction
PCPS: Percutaneous cardiopulmonary support
SAVR: Surgical aortic valve replacement
TAVI: Transcatheter aortic valve implantation
